# Akt and mTOR in B Cell Activation and Differentiation

**DOI:** 10.3389/fimmu.2012.00228

**Published:** 2012-08-06

**Authors:** Jose J. Limon, David A. Fruman

**Affiliations:** ^1^Department of Molecular Biology and Biochemistry, Institute for Immunology, University of California IrvineIrvine, CA, USA

**Keywords:** B cells, proliferation, differentiation, antibody, PI3K, Akt, mTOR, kinase

## Abstract

Activation of phosphoinositide 3-kinase (PI3K) is required for B cell proliferation and survival. PI3K signaling also controls key aspects of B cell differentiation. Upon engagement of the B cell receptor (BCR), PI3K activation promotes Ca^2+^ mobilization and activation of NFκB-dependent transcription, events which are essential for B cell proliferation. PI3K also initiates a distinct signaling pathway involving the Akt and mTOR serine/threonine kinases. It has been generally assumed that activation of Akt and mTOR downstream of PI3K is essential for B cell function. However, Akt and mTOR have complex roles in B cell fate decisions and suppression of this pathway can enhance certain B cell responses while repressing others. In this review we will discuss genetic and pharmacological studies of Akt and mTOR function in normal B cells, and in malignancies of B cell origin.

## Overview of PI3K Effectors in B Cells

Phosphoinositide 3-kinases (PI3Ks) are a family of lipid kinase enzymes that produce 3′-phosphorylated phosphoinositides (Okkenhaug and Fruman, 2010; Vanhaesebroeck et al., 2010; So and Fruman, 2012). These lipids act as second messengers to redirect cytoplasmic proteins to cellular membranes. The production of phosphatidylinositol-3,4,5-trisphosphate (PIP_3_) by class I PI3Ks is a shared response downstream of a variety of receptors in all mammalian cell types. In B cells, class I PI3K activation is initiated when the B cell receptor (BCR) recognizes antigen and is augmented by CD19, a component of the B cell co-receptor. Class I PI3K activity is necessary for BCR-dependent proliferation and is sufficient for BCR-dependent tonic survival signaling. T cell-derived cytokines including interleukin-4 (IL-4) augment and sustain PI3K activity during B cell growth and clonal expansion. Toll-like receptor (TLR) engagement, chemokine signaling, and cytokines (e.g., BAFF) also trigger class I PI3K activation. The central role of PI3K activation in B cell function has prompted detailed studies of signaling mechanisms downstream of PI3K.

**Figure 1:**
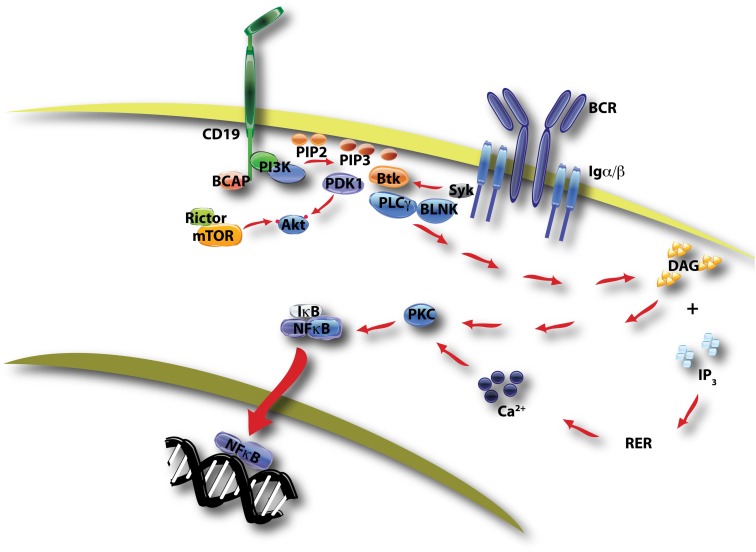
**This diagram of BCR/CD19-initiated signaling shows two key outcomes of PI3K activation**. PIP3 production by class IA PI3K promotes formation of a signalosome containing Btk, BLNK, and PLCγ, leading to hydrolysis of PI-4,5-P2 by PLCγ. The products of this reaction, DAG and IP3, promote the activation of PKC and ultimately the nuclear translocation of NFκB transcription factors. This pathway is essential for proliferation following antigen recognition. A second outcome of PIP3 production is the membrane recruitment of PDK-1 and Akt, with subsequent phosphorylation of Akt on two distinct sites by PDK-1 and mTORC2 (mTOR in complex with rictor, see Figure 2).

Proteins recruited to the membrane through binding to PI3K lipid products are generally termed PI3K effectors (Fruman, 2004; Lemmon, 2008). Most effectors of class I PI3K have a pleckstrin homology (PH) domain that binds selectively to PIP_3_ and/or phosphatidylinositol-3,4-bisphosphate (PtdIns-3,4-P_2_). Btk and Tec are closely related protein tyrosine kinases whose PH domains bind with high affinity to PIP_3_. These kinases function in a large protein assembly called the BCR signalosome, whose primary output is activation of phospholipase-C-gamma (PLCγ), leading to production of diacylglycerol (DAG) and inositol-1,4,5-trisphosphate (IP_3_; Fruman et al., 2000; Fruman, 2004). Together these second messengers promote Ca^2+^ mobilization, protein kinase C (PKC) activation, and ultimately the nuclear translocation of NFκB transcription factors to drive B cell proliferation (Figure 1). Loss of Btk or blockade of its binding to PIP_3_ reduces the Ca^2+^ response, diminishes NFκB activation, and prevents productive B cell activation. Conversely, forced activation of PKC or NFκB restores BCR-dependent responses in B cells lacking PI3K or Btk function. These findings suggest that signalosome assembly and NFκB activation are the most important functional outcomes of PI3K signaling during BCR-stimulated B cell activation. Moreover, genetic inactivation of class I PI3K in both humans and mice causes a B cell deficiency similar to the Btk-loss phenotype (Fruman et al., 1999; Suzuki et al., 1999; Conley et al., 2012). Apart from the Ca^2+^ signalosome, PI3K activation triggers the membrane recruitment of additional protein assemblies that might also have key functions.

Two PH domain-containing proteins linked to PI3K activity in all cells, including B cells, are the serine/threonine kinases Akt and phosphoinositide-dependent kinase-1 (PDK-1; Fayard et al., 2010). The three related Akt kinases (Akt1, Akt2, Akt3; also known as PKBα,β,γ) each contain a threonine residue in the activation loop (T308 in Akt1) that is phosphorylated by PDK-1 in a manner dependent on PI3K activity (Figure 1). Subsequent phosphorylation of a serine in a hydrophobic motif (S473 in Akt1) by a distinct kinase is required for maximal Akt activation. All three Akt isoforms are expressed in B lineage cells and their functions appear to be partially redundant (Calamito et al., 2010). Of the many Akt substrates reported, the Forkhead Box, Subgroup O (FOXO) transcription factors are of particular importance for B cell biology as discussed below. Akt-mediated FOXO phosphorylation suppresses their transcriptional activity and causes their nuclear export and sequestration or degradation (Burgering, 2008).

**Figure 2:**
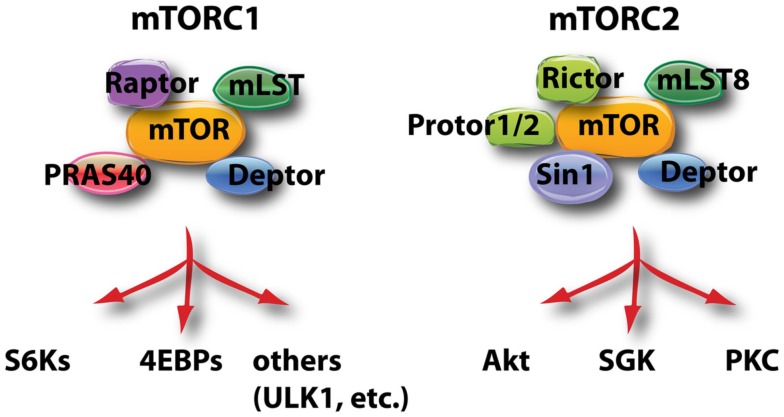
**This schematic diagram shows the components of mTORC1 and mTORC2, and the most well known substrates of the two complexes**. ULK1 (Unc51-like kinase-1) is involved in the control of autophagy.

The mTOR kinase is encoded by a single gene in mammals but is the active enzyme in two distinct multi-protein complexes called mTORC1 and mTORC2 (Figure 2; Laplante and Sabatini, 2012). mTORC1 is defined by the raptor subunit and mTORC2 by the rictor subunit. The main function of mTORC1 is to sense nutrients and mitogenic signals; when conditions are favorable, mTORC1 triggers biosynthetic pathways essential for cell growth and proliferation. Akt can promote mTORC1 activation through several mechanisms, as described in more detail below. mTORC2 activity can be stimulated by growth factors but is nutrient-independent. The best-described function of mTORC2 is to phosphorylate Akt on S473. However, other kinases can phosphorylate this site in certain conditions (Fayard et al., 2010) and mTORC2 has other important substrates as well, including serum- and glucocorticoid-induced kinase (SGK) and PKC as shown in Figure 2. SGK family members have some overlapping properties with Akt isoforms, for example the ability to phosphorylate FOXO transcription factors (Brunet et al., 2001).

Overall, the signaling network defined by Akt and the two mTOR complexes is a central driver of cell growth, metabolism, and proliferation, and the activity of this network is elevated in nearly all human cancers (Engelman et al., 2006; Liu et al., 2009). Consequently, there has been great interest in targeting the pathway for therapeutic benefit in cancer. In parallel, Akt and mTOR signaling has been actively studied in the context of normal lymphocyte function (So and Fruman, 2012). Indeed, mTOR was first discovered and named as the target of rapamycin, an immunosuppressive drug that is now used clinically to prevent organ transplant rejection (Guertin and Sabatini, 2009). Rapamycin suppresses T cell proliferation, promotes regulatory T cell differentiation, and modulates the function of innate immune cells. In this review we describe our current knowledge of Akt and mTOR functions in B lymphocytes.

## The Akt-FOXO Axis in B Cell Development, Activation, and Differentiation

Akt was first defined as a key PI3K effector in 1995 (Franke et al., 1995). Within a few years, several groups had shown that Akt is recruited to the membrane and activated downstream of the BCR and CD19, in a manner dependent on PI3K (Aman et al., 1998; Astoul et al., 1999; Pogue et al., 2000; Otero et al., 2001). Subsequently, we reported that BCR signaling through PI3K downregulates expression of FOXO target genes *Rbl2* and *Ccng2* (Fruman et al., 2002; Yusuf et al., 2004). These genes encode the proteins p130 and cyclin G2, both implicated in cell cycle arrest in non-lymphoid cells (Kops et al., 2002; Martinez-Gac et al., 2004). Consistent with a role for FOXO factors in opposing cell cycle progression, Akt-dependent inactivation of FOXO transcription factors is important for optimal B cell proliferation in response to lipopolysaccharide (LPS; Yusuf et al., 2004). It is likely that Akt has many other substrates that play key roles in B cell biology. However, the Akt-FOXO axis has emerged as a key control point for various aspects of B cell function.

**Figure 3:**
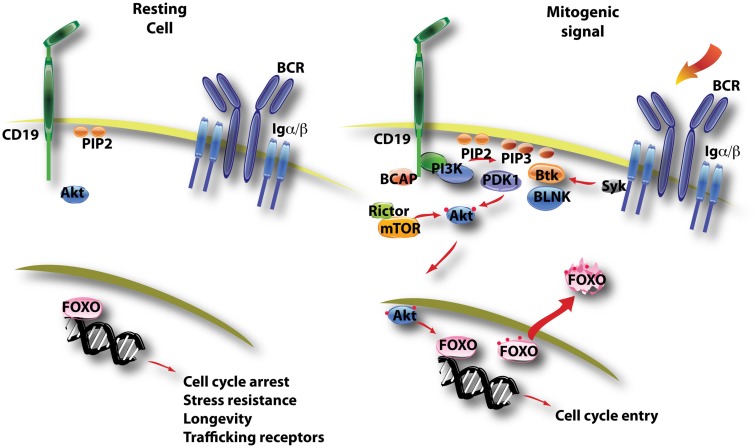
**This diagram illustrates the control of FOXO function by PI3K/Akt activation**. In resting B cells, FOXO factors are mainly nuclear and direct a gene expression program favoring quiescence (cell cycle arrest, longevity) and recirculation (trafficking through blood and lymphoid tissue). B cell activation triggers PI3K/Akt activity, and active Akt enters the nucleus to phosphorylate FOXO proteins on several conserved sites (noted by red dots). This leads to nuclear export of FOXO factors that are then sequestered and/or degraded in the cytoplasm.

FOXO transcription factors (FOXO1, FOXO3a, FOXO4, FOXO6) are an evolutionarily conserved family of proteins whose activity is tightly controlled by growth factors (Burgering, 2008). In the absence of mitogenic signals, FOXO proteins are mainly nuclear and direct a transcriptional program that blocks cell cycle progression and promotes stress resistance and longevity (Figure 3). FOXO factors can also promote expression of pro-apoptotic genes (Fu and Tindall, 2008). Growth factor receptor signaling inactivates FOXO through Akt-dependent phosphorylation on three conserved serine or threonine residues. These phosphorylation events trigger the release of FOXO from DNA, nuclear export, and sequestration or degradation in the cytoplasm (Figure 3). Some of the consensus sites for Akt phosphorylation are also substrates for SGKs, whose activity is not as tightly coupled to PI3K signaling (Brunet et al., 2001). Also, FOXO function is regulated further by acetylation and by the status of cooperating transcription factors (Calnan and Brunet, 2008). Nevertheless, PI3K/Akt activation plays a dominant role in regulation of FOXO activity. Both FOXO1 and FOXO3 are controlled by Akt-mediated phosphorylation and both isoforms are expressed in B lineage cells (Dengler et al., 2008; Hinman et al., 2009; Lin et al., 2010).

Foxo1 is an essential component of a transcription factor network in pro-B cells that also includes E2A and EBF1 (Lin et al., 2010). This study showed that E2A binds to regulatory elements upstream of the *Foxo1* gene, and that FOXO1 protein functions together with E2A and EBF1 to induce transcription of the *Pax5* gene to drive B cell commitment. An unanswered question is how FOXO1 retains a required nuclear function in B cell progenitors, which are continuously exposed to cytokines and other signals that activate PI3K/Akt signaling. Gene knockout studies have confirmed that the *Foxo1* gene is essential for proper B cell development. Using mice with a conditional allele of *Foxo1*, Rickert and colleagues analyzed FOXO1 function at various stages using different Cre deleter strains (Dengler et al., 2008). Deletion at an early stage using *Mb1*-Cre causes a partial block at the pro-B cell stage that can be attributed to impaired expression of the interleukin-7 receptor. Deletion in late pro-B cells using *Cd19*-Cre causes a block at the pre-B cell stage owing to reduced expression of the recombination activating genes (*Rag-1* and *Rag-2*). Similarly, FOXO3a-deficient mice have reduced numbers of pre-B cells (Hinman et al., 2009). Others have shown that *Rag* genes are direct targets of FOXO1 and FOXO3a (Amin and Schlissel, 2008; Herzog et al., 2008). Successful rearrangement of heavy and light chain genes produces functional pre-BCR and mature BCR expression, and it is likely that basal (tonic) signaling through these receptors acts through PI3K and Akt to suppress FOXO function and turn off *Rag* gene expression to achieve allelic exclusion. Consistent with this model, mice lacking PI3K function in pre-B cells have an elevated fraction of cells with two rearranged heavy chain alleles (Ramadani et al., 2010).

PI3K also regulates the fate of immature B cells. Successful light chain gene recombination at the pre-B cell stage results in expression of surface immunoglobulin (the mature BCR) and transition to the immature B cell stage. Tonic signaling through the BCR is required to extinguish *Rag* gene expression, exit the pre-B cell stage and positively select immature B cells for further differentiation (Tze et al., 2005). Three reports showed that PI3K activity is required for basal BCR signals at the immature B cell stage, with a selective role for p85α and p110δ isoforms (Tze et al., 2005; Llorian et al., 2007; Verkoczy et al., 2007). However, the available data indicate that PI3K suppresses *Rag* gene expression at this stage via phospholipase C-γ rather than through the Akt-FOXO axis (Verkoczy et al., 2007).

**Figure 4:**
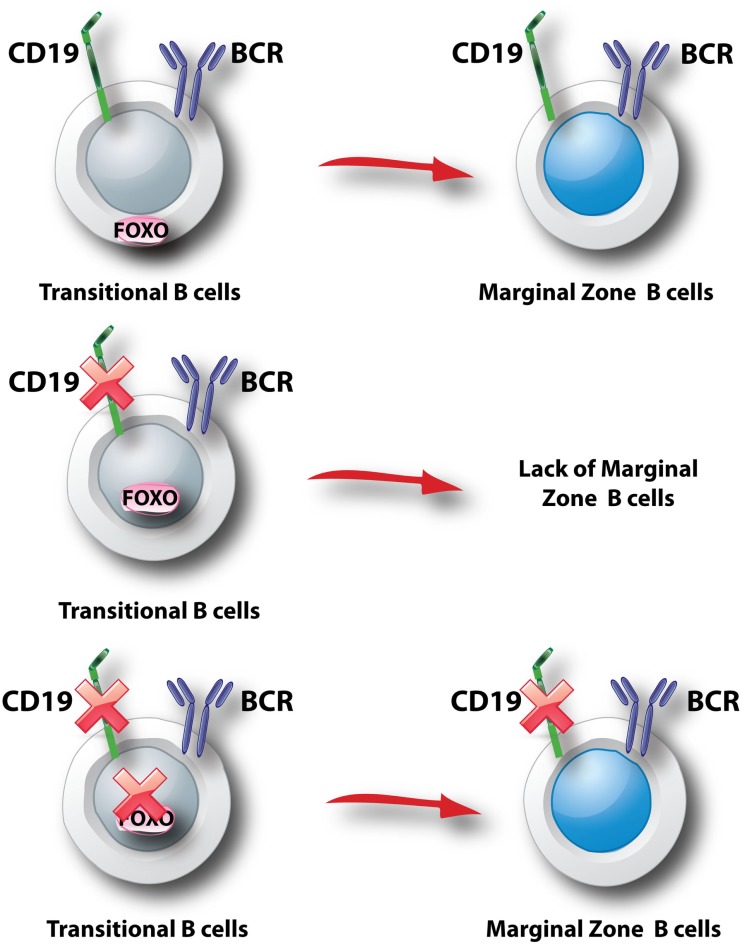
**Model for control of marginal zone B cell development**. It is thought that transitional B cell selection into the MZ lineage is driven by interactions of the BCR with self antigens (Wen et al., 2005). In addition, CD19 associates with BCR signalosomes and plays a key role in recruiting PI3K to the membrane and activating Akt following BCR engagement (Otero et al., 2001; Wang et al., 2002; Aiba et al., 2008; Depoil et al., 2008). Genetic studies support the model that commitment to the MZ lineage requires a signal initiated by CD19 and propagated by PI3K (p85α/p110δ) to Akt (not shown in this figure). This signal blocks nuclear entry of FOXO (top panel). In the absence of CD19, the signal is interrupted and Foxo1 can program a block to MZ development (middle panel). In the absence of Foxo1, CD19 is dispensable for MZ B cell development (bottom panel).

Deletion of *Foxo1* with *Cd19*-Cre does not fully block the formation of mature B cells (Dengler et al., 2008; Chen et al., 2010). However, peripheral lymphoid organs from these mice show aberrant representation of B cell subsets. There is a large fraction of B220^+^ cells lacking surface Ig (Dengler et al., 2008; Chen et al., 2010), which may represent B cell progenitors escaping the bone marrow due to altered expression of trafficking receptors. There is also a significant increase in the marginal zone (MZ) B cell subset (Dengler et al., 2008; Chen et al., 2010). The opposite phenotype, a reduced MZ B cell compartment, is observed upon deletion of PI3K catalytic (p110δ) or regulatory (p85α) subunits or inactivation of both Akt1 and Akt2 (Okkenhaug et al., 2002; Suzuki et al., 2003b; Donahue et al., 2004; Calamito et al., 2010). A similar loss of MZ B cells occurs in mice lacking CD19, but not in mice lacking Btk (Donahue et al., 2004). Furthermore, the MZ B cell defect in CD19-deficient mice can be reversed by combined deletion of *Foxo1* (Chen et al., 2010). Together these observations suggest that commitment to the MZ fate in transitional B cells is driven by CD19 signaling through PI3K and Akt to inactivate FOXO factors (Figure 4). The FOXO target genes that restrain MZ B cell commitment have not been established. The Notch signaling pathway can promote MZ B cell development even in the absence of CD19 (Hampel et al., 2011), suggesting that FOXO proteins might oppose Notch signaling. However, in other cellular systems Notch and FOXO were shown to cooperate (Kitamura et al., 2007).

In the T cell lineage, a major function of FOXO proteins is to maintain expression of the lymph node homing receptor CD62L and other trafficking receptors necessary for proper recirculation of quiescent cells through blood and lymphoid tissues (Fabre et al., 2008; Kerdiles et al., 2009; Ouyang et al., 2009). Similarly, deletion of *Foxo1* in late transitional B cells (using *Cd21*-Cre) impairs CD62L expression on mature B cells (Dengler et al., 2008; Figure 3). This results in altered homing with fewer B cells detected in the lymph nodes (Dengler et al., 2008; Chen et al., 2010). In wild-type B cells, BCR-dependent downregulation of CD62L is partially dependent on PI3K activity (Hess et al., 2004). FOXO activity probably controls CD62L transcription indirectly via Krüppel-like factors (KLFs) in B cells, as in T cells (Hart et al., 2012).

Mature B cells lacking FOXO1 show reduced surface expression of the BCR and significantly reduced BCR signaling responses including Ca^2+^ mobilization and phosphorylation of Akt and ERK (Dengler et al., 2008). The mechanism for signal attenuation in the absence of FOXO1 has not been established. However, there is a potential connection to cancer cell lines in which PI3K/Akt inhibition leads to FOXO-dependent upregulation of receptor tyrosine kinase expression and function (Hay, 2011). Therefore, FOXO1 in B cells might maintain expression of signaling proteins necessary for activation, such that BCR-PI3K-Akt signaling would inactivate FOXO1 to trigger a built-in negative feedback mechanism. BCR signaling through PI3K also suppresses *Foxo1* expression at the transcriptional level (Hinman et al., 2007).

At the mature B cell stage, exposure to the cytokine BAFF and continuous expression of the surface BCR are essential to maintain survival (Lam et al., 1997; Schiemann et al., 2001). Mouse genetic models have shown that PI3K activity is both necessary and sufficient to maintain survival of mature peripheral B cells (Srinivasan et al., 2009; Ramadani et al., 2010). Rescue of BCR-negative cells by expression of constitutively active PI3K or deletion of PTEN correlates with low but detectable levels of Akt phosphorylation (Srinivasan et al., 2009). It is likely that Akt activity has an important function in peripheral B cell survival, as B cells lacking both Akt1 and Akt2 show reduced fitness compared to wild-type in a competitive repopulation assay (Calamito et al., 2010). In addition, deletion of *Foxo1* partially rescues survival of BCR-negative peripheral B cells, though the rescued cells have low CD62L expression and do not accumulate in lymph nodes (Srinivasan et al., 2009).

**Figure 5:**
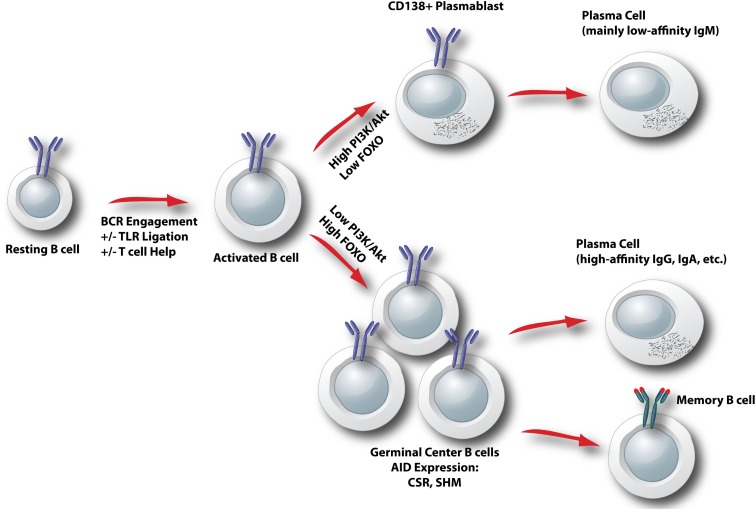
**Model for control of activated B cell differentiation fate by the Akt-FOXO axis**. In activated B cells, the degree of ongoing PI3K/Akt signaling determines the relative nuclear activity of FOXO. When PI3K/Akt is high, FOXO activity is low and plasmablast differentiation is favored. When PI3K/Akt is low, FOXO activity is elevated and a germinal center B cell fate is programmed. CSR, class switch recombination; SHM, somatic hypermutation.

Following B cell clonal selection by antigen, activated B cells commit to one of two distinct differentiation pathways. Some cells undergo rapid differentiation into antibody-secreting plasma cells that secrete mostly IgM antibodies of low affinity. Other cells commit to the germinal center (GC) fate, and surviving clones emerge 1–2 weeks later as memory or plasma cells making high affinity class switch antibodies (Figure 5). There is accumulating evidence that the choice between rapid plasmablast differentiation versus GC entry is determined by the degree of PI3K-Akt signaling, and thus by the level of FOXO activity (Figure 5). Elevation of PI3K-Akt signaling through loss of PTEN increases antibody-secreting cell (ASC) differentiation and strongly suppresses class switch recombination (CSR; Suzuki et al., 2003a; Omori et al., 2006). The mechanism is directly linked to the Akt-FOXO axis, since CSR can be restored in PTEN-deficient cells by expression of constitutively active FOXO proteins (Omori et al., 2006). Furthermore, mature B cells lacking *Foxo1* have severe defects in class switching due to a failure to upregulate the gene *Aicda* encoding the AID mutator protein (Dengler et al., 2008). In contrast, PI3K inhibitors enhance AID expression and class switching and oppose ASC differentiation *in vitro* (Omori et al., 2006). Genetic or pharmacological blockade of the p110δ isoform of class I PI3K specifically enhances the production of IgE (Zhang et al., 2008), but this appears to be independent of Akt (Zhang et al., 2012). It is important to note that whereas PI3K is essential for proliferation triggered by BCR engagement, this requirement can be bypassed when B cells are activated through other receptors such as TLRs, CD40, and IL-4. Indeed, B cells stimulated with CD40L + IL-4 proliferate in the presence of PI3K inhibitors and it is these conditions that have revealed the role for PI3K-Akt signaling in opposing CSR (Fruman et al., 1999; Omori et al., 2006). Similarly, LPS-stimulated B cells maintain the ability to proliferate and show elevated plasmablast differentiation in the presence of PI3K inhibitors (Omori et al., 2006). Further complicating the issue, class I PI3K plays a positive role in the ability of BCR engagement to enhance CSR initiated by TLR ligands (Pone et al., 2012). This phenomenon is linked to NFκB activation, and might be independent of the Akt-FOXO axis.

In the context of T cell-dependent antibody responses, PI3K inhibition can limit GC responses by suppressing the differentiation and function of T follicular helper (Tfh) cells (Rolf et al., 2010). PI3K function in Tfh cells might depend on Akt activation and FOXO inhibition, since deletion of *Foxo1* in T cells enhances Tfh differentiation and expands GCs (Kerdiles et al., 2010). This phenotype might be an indirect effect of reduced Treg function in mice with *Foxo1*-deficient T cells (Kerdiles et al., 2010), since Tregs suppress GC responses (Alexander et al., 2011; Chung et al., 2011; Linterman et al., 2011; Wollenberg et al., 2011). Nevertheless, at a global level the Akt-FOXO axis directs opposing outcomes in T and B cells with respect to GC differentiation. In T cells, FOXO loss enhances GCs whereas in B cells FOXO loss reduces GC differentiation. Therefore it will be interesting to determine how inhibitors targeting the PI3K/Akt network at different levels will affect antibody responses to different types of pathogens and vaccines.

## Rapamycin and the Two mTOR Complexes in B Cells

mTORC1 is composed of the mTOR enzyme, raptor, PRAS40, mLST8, and DEPTOR (Figure 2; Laplante and Sabatini, 2012). Raptor is essential for phosphorylation of mTORC1 substrates, whereas PRAS40 and DEPTOR suppress kinase activity. The mechanisms of mTORC1 activation and repression are complex and have been worked out mainly in non-lymphoid cell types. Nutrients sustain mTORC1 activity through amino acid-dependent recruitment to a complex of proteins containing Rag GTPases, termed the Ragulator (Sancak et al., 2010), whereas glucose metabolism prevents the activation of AMP kinase, a negative regulator of mTORC1. However, nutrient availability alone is not sufficient to fully activate mTORC1; growth factor inputs (antigen receptors and costimulatory receptors in lymphocytes) are also required. Mitogenic signals activate mTORC1 through several mechanisms. First, Akt and/or Erk phosphorylate and suppress the tuberous sclerosis complex (TSC1/TSC2), relieving its GAP activity toward the Rheb small GTPase that is an essential upstream activator of mTORC1. Akt can also phosphorylate and inactivate PRAS40, one of the intrinsic negative regulators of the mTORC1 complex. Production of phosphatidic acid also occurs during cell activation and this can contribute to mTORC1 activation. One of the surprising findings of the last several years has been that mTORC1 activation in lymphocytes can occur even when PI3K/Akt activity is undetectable. This was first observed in B cell lymphoma lines (Wlodarski et al., 2005). Later we reported that the mechanism of mTORC1 activation in normal mature B cells depends on the stimulus (Donahue and Fruman, 2007). BCR-dependent mTORC1 activation is PI3K-dependent whereas basal and LPS-dependent mTORC1 activity is partly PI3K-independent. Similarly, mTORC1 activity is sustained in activated T cells lacking detectable PI3K/Akt activity (Deane et al., 2007). Other Akt-independent inputs are likely to regulate downstream mTORC1 substrates in lymphocytes, as has been shown for the S6 ribosomal protein in CD8 T cells (Salmond et al., 2009).

mTORC2 is composed of three components essential for activity (rictor, Sin1, mLST8) along with regulatory components DEPTOR and PROTOR (Figure 2; Laplante and Sabatini, 2012). Based on studies mainly done in non-lymphoid cell types, current models indicate that mTORC2 has some basal activity in cells and phosphorylates sites in Akt and certain PKC isoforms that are important for folding and stability (Cybulski and Hall, 2009). Growth factor stimulation promotes increased activity of mTORC2 through association with ribosomes (Zinzalla et al., 2011), and subsequent mTORC2-dependent phosphorylation of the hydrophobic motif of various AGC family kinases – most notably the S473 site on Akt. Thus, mTORC2 acts upstream of Akt whereas mTORC1 acts downstream of Akt. Recent data indicate that PI3K lipid products stimulate mTORC2 activity (Gan et al., 2011; Tato et al., 2011; Shanmugasundaram et al., 2012); the degree of basal mTORC2 activity maintained in PI3K-deficient lymphocytes is not known.

Rapamycin was first identified in a screen for natural products with anti-fungal activity but was later shown to suppress lymphocyte proliferation (Sehgal et al., 1975; Martel et al., 1977; Calne et al., 1989). Although most studies of rapamycin mechanism have focused on T cells, it is often overlooked that rapamycin suppresses B cell proliferation more completely. Three papers in the early 1990s established that rapamycin has profound effects on B cell proliferation and differentiation (Wicker et al., 1990; Kay et al., 1991; Aagaard-Tillery and Jelinek, 1994). Pretreatment with rapamycin completely blocked murine B cell proliferation induced by anti-IgM (±IL-4), and reduced by 50–60% the response to LPS (Wicker et al., 1990; Kay et al., 1991). Rapamycin prevented B cell growth but did not impact survival (Wicker et al., 1990). In human B lymphocytes stimulated with the polyclonal activator *S. aureus*, rapamycin reduced cell proliferation by 60–80% and completely blocked differentiation into ASCs (Aagaard-Tillery and Jelinek, 1994). Notably, rapamycin suppressed T cell receptor (TCR)-dependent proliferation of CD4 T cells to a lesser extent than BCR-dependent cell proliferation (Kay et al., 1991), and more recent work showed that genetic deletion of mTOR in T cells delays but does not block clonal expansion (Delgoffe et al., 2009). Rapamycin does not strongly suppress CD8 T cell expansion (Slavik et al., 2001) and actually enhances generation of memory CD8 cells (Araki et al., 2009). Therefore, the profound effect on BCR-driven proliferation is unusual and highlights that rapamycin could be an effective approach for treatment of B cell-driven autoimmune diseases. Indeed, rapamycin reduces pathogenic antibody accumulation and ameliorates disease in mouse models of lupus (Warner et al., 1994; Lui et al., 2008).

The mechanisms by which rapamycin blocks B cell cycle entry and differentiation remain unclear. mTORC1 has many substrates, of which the most well-studied are the ribosomal S6 kinases (S6K1 and S6K2) and the eIF4E binding proteins (4EBPs; Figure 2; Laplante and Sabatini, 2012). S6Ks promote protein and lipid synthesis and their activity is completely dependent on mTORC1-mediated phosphorylation (Magnuson et al., 2012). In contrast, the phosphorylation of 4EBPs by mTORC1 is an inhibitory event that blocks the ability of 4EBPs to suppress eIF4E function in cap-dependent translation (Silvera et al., 2010). Overall, the phosphorylation of S6Ks and 4EBPs along with the suppression of autophagy by active mTORC1 are essential for cell growth in preparation for division. Phosphorylation of S6Ks and 4EBPs occurs rapidly following BCR engagement (Donahue and Fruman, 2007), but whether these signals are required for successful B cell growth and proliferation has not been determined. Rapamycin strongly suppresses S6K phosphorylation in all cell types, but has limited and variable effects on 4EBP phosphorylation (Choo et al., 2008; Thoreen and Sabatini, 2009). Rapamycin does not acutely inhibit mTORC2 but chronic treatment with rapamycin can suppress mTORC2 assembly in some cell types, including lymphocytes (Sarbassov et al., 2006; Lazorchak et al., 2010; Delgoffe et al., 2011). Further work is necessary to establish whether inhibition of mTORC1 and/or mTORC2 underlies the effects of rapamycin in B cells. It is also possible that rapamycin has targets other than mTOR in lymphocytes, or that a kinase-independent scaffolding function of mTOR is disrupted upon rapamycin exposure.

Surprisingly little is known about the unique functions of the mTOR complexes in B cells. Rapamycin is not an optimal tool for addressing this question, since as noted above the compound is only a partial inhibitor of mTORC1 (Choo et al., 2008; Thoreen and Sabatini, 2009) and its effects on mTORC2 are dependent on concentration and time of treatment (Sarbassov et al., 2006; Lazorchak et al., 2010; Delgoffe et al., 2011). To date there have been no publications describing the phenotype of mature B cells lacking essential components of mTORC1 or mTORC2. One report described a B cell-specific knockout of Sin1 but the analysis focused on progenitor B cell development in the bone marrow (Lazorchak et al., 2010). Interestingly, Sin1-deficient cells showed elevated expression of IL-7 receptor and RAG proteins, consistent with increased FOXO activity when mTORC2-Akt (and SGK) signaling is reduced. In Sin1-deficient pre-B cells cultured from bone marrow *in vitro*, expression of constitutively active Akt2 led to FOXO1 phosphorylation and reduced RAG expression (Lazorchak et al., 2010). Akt2 might not be essential for FOXO regulation *in vivo*, as B cell development is not impaired in mice lacking Akt2 or in chimeric mice lacking both Akt1 and Akt2 in B cell progenitors (Calamito et al., 2010). Notably, prolonged rapamycin treatment suppressed mTORC2-dependent Akt phosphorylation leading to elevated expression of FOXO1 and RAG proteins (Lazorchak et al., 2010).

Although B cells lacking mTORC1 function have not yet been described, one study analyzed the consequences of B cell-specific loss of the negative regulator TSC1 (Benhamron and Tirosh, 2011). As expected, mTORC1 activity was increased as judged by phosphorylation of S6 protein downstream of S6Ks. A striking phenotype was a reduced percentage of MZ B cells. The most likely explanation is that elevated mTORC1 activity in transitional B cells engages negative feedback loops that reduce activity of upstream PI3K/Akt signaling. Well-established negative feedback mechanisms include S6K-dependent phosphorylation of insulin receptor substrate (IRS) proteins and mTORC1-dependent phosphorylation of the adaptor protein Grb10 (Yea and Fruman, 2011; Laplante and Sabatini, 2012). As discussed earlier, reduced PI3K/Akt activity is known to diminish MZ B cell development. However, the status of PI3K/Akt signaling in TSC1-deficient B cells was not assessed in this study.

An interesting report described the phenotype of mice with a hypomorphic allele of *Mtor* that reduces mTOR protein expression and diminishes the activity of both mTORC1 and mTORC2 (Zhang et al., 2011). These mice have a greatly reduced number of peripheral B cells and a partial block in early development at the large pre-B cell stage. Whether this block is associated with altered FOXO function or RAG expression was not investigated. B cells with reduced mTOR expression have greatly impaired proliferative responses to anti-IgM or anti-CD40, whereas the LPS response is largely intact. Curiously, B cells from these mice display elevated phosphorylation of Akt on S473 following LPS stimulation. This correlates with increased expression of DNA-PK, an alternative kinase for Akt-S473, and can be reduced by a selective DNA-PK inhibitor. These findings highlight that the effect of mTOR inhibition on Akt-S473 phosphorylation is highly context-dependent. Whether chronic treatment with mTOR catalytic inhibitors would also lead to DNA-PK upregulation in B cells is not known.

## Akt and mTOR in B Cell Malignancies

Activation of the PI3K/Akt/mTOR signaling network is a common feature of most human cancers (Engelman et al., 2006; Liu et al., 2009; Hanahan and Weinberg, 2011). Malignancies of B cell origin are no exception. Many groups have documented high basal levels of Akt and mTOR activation in B cell leukemias, B cell lymphomas, and multiple myeloma (MM) cells. For example, the BCR-ABL oncoprotein strongly activates PI3K/Akt signaling and mTOR activity in Philadelphia Chromosome-positive (Ph^+^) leukemias (Skorski et al., 1997; Kharas et al., 2008; Janes et al., 2010). mTOR is activated in a Syk-dependent manner in follicular lymphoma cells (Leseux et al., 2006; Fruchon et al., 2012). In diffuse large B cell lymphoma (DLBCL), the microRNA miR-155 is often overexpressed leading to reduced amounts of the SHIP phosphatase that can dephosphorylate the 5’-phosphate of PIP_3_ (Pedersen et al., 2009). The activated B cell subset of DLBCL displays constitutive Akt signaling through chronic active BCR signaling (Davis et al., 2010). Interestingly, a fraction of MM tumors shows elevated expression of DEPTOR, an endogenous inhibitor of mTORC1 and mTORC2 (Peterson et al., 2009). In this case, DEPTOR appears to be important for limiting mTORC1-dependent negative feedback on PI3K/Akt activity.

The central role of PI3K/Akt/mTOR signaling in B cell neoplasms has led many investigators to test the efficacy of small molecule inhibitors of this network. Proof-of-concept was provided first by clinical trials showing significant responses to temsirolimus (CCI-779; an orally active rapamycin analog) in patients with mantle cell lymphoma (Hess et al., 2009). Such rapalogs have shown generally more limited success in other clinical trials of leukemia, lymphoma, and myeloma (Kelly et al., 2011). ATP-competitive compounds that target of the active site of mTOR show more promise. In preclinical studies, dual-targeted agents that directly inhibit both PI3K and mTOR (e.g., PI-103, NVP-BEZ235) have shown efficacy in Ph^+^ pre-B cell acute leukemia (Kharas et al., 2008), chronic lymphocytic leukemia (CLL; Niedermeier et al., 2009), various B cell lymphomas (Bhatt et al., 2010; Bhende et al., 2010), and MM (Baumann et al., 2009).

Selective mTOR kinase inhibitors appear to be as effective as panPI3K/mTOR inhibitors in models of Ph^+^ leukemia (Carayol et al., 2010; Janes et al., 2010) and MM (Maiso et al., 2011), with lesser toxicity (Janes et al., 2010). However, there might be some B cell malignancies in which dual PI3K/mTOR inhibition or PI3K inhibition alone might be most effective. Recent studies have revealed that selective inhibition of the p110δ isoform of PI3K produces remarkable clinical responses in CLL patients (Fruman and Rommel, 2011). Similarly, Btk inhibitors in clinical development have shown great promise in clinical trials of CLL treatment (Winer et al., 2012). Thus, the connection of PI3K and Btk is not limited to BCR-mediated activation of normal B cells, but seems to represent a key signaling axis for CLL cell proliferation, survival, and migration. Although antibody-mediated B cell depletion (anti-CD20; rituximab) often provides benefit for the treatment of B cell malignancies, PI3K/Btk-targeted small molecules might have some advantages. Such agents would be more rapidly reversible than long-lived antibodies upon cessation of treatment, allowing prompt resolution of adverse immunosuppressive effects. Small molecule orally active compounds might also be more convenient and less expensive to administer. It is also possible that PI3K/Btk inhibitors will be useful as adjuncts to rituximab, as suggested by preliminary reports of combination trials in non-Hodgkin’s lymphoma (Fruman and Rommel, 2011; Winer et al., 2012). Ultimately, the optimal PI3K/mTOR inhibitors and combinations for different malignancies will require careful comparison of efficacy and tolerability in clinical trials.

## Summary and Future Directions

In B cells activated through BCR crosslinking, treatment with either PI3K inhibitors or rapamycin profoundly blocks B cell proliferation. This suggests a direct function of mTOR downstream of PI3K in BCR signaling. However, subsequent studies of PI3K, Akt, and mTOR signaling in B cells have led to a number of surprises. Whereas rapamycin completely blocks differentiation of B cells stimulated with TLR ligands or T cell-derived helper factors (i.e., CD40L + IL-4), PI3K inhibition has the distinct effect of enhancing CSR while suppressing terminal differentiation to plasma cells. Deletion of *Foxo1*, which might have been predicted to lower the threshold for B cell activation, actually attenuates B cell proliferation and differentiation. We propose a model in which two key downstream PI3K effector arms in B cells have distinct functions. In simple terms, the Ca^2+^ signalosome drives proliferation, whereas the Akt-FOXO axis controls differentiation. Following antigen recognition, BCR signaling through PI3K leads to signalosome assembly to drive cell cycle progression primarily through NFκB activation (Figure 1). The subsequent differentiation path of the activated B cell is controlled by the kinetics and magnitude of PI3K activation through the BCR and other signals including TLR engagement and T cell help (Figure 5). High PI3K/Akt activity suppresses FOXO function to promote rapid production of plasma cells secreting mainly IgM. Low PI3K/Akt activity allows FOXO function to be re-established, and programs the cell to express AID and commit to the GC B cell fate. This mechanism makes sense in that it allows the host to tailor the antibody response to the antigen. When there is a high affinity or abundant antigen, the goal is to make antibodies quickly. This is achieved through sustained PI3K/Akt signaling that drives plasma cell differentiation. When the antigen is of low affinity or not abundant, eradication of the antigen requires high affinity class switched antibodies. This would be achieved because the reduced antigen-derived signals limit PI3K/Akt activity, allowing FOXO factors to program the GC B cell fate.

A question that arises from this model is why mTOR inhibition with rapamycin is such a potent inhibitor of both B cell proliferation and differentiation, regardless of the stimulus. This finding suggests an essential function of mTORC1 in these processes. One possibility is that mTORC1 activity is required in B cells and that sufficient mTORC1 function is maintained even when PI3K/Akt activity is suppressed. However, the mTORC1 substrates whose function is essential in B cells have not been established. Future work should investigate the functions of S6Ks, 4EBPs, and other mTORC1 substrates in B cells. Complicating matters, we have shown that ATP-competitive mTOR kinase inhibitors have distinct effects on B cells compared to rapamycin. At concentrations that strongly suppress phosphorylation of mTORC1 and mTORC2 substrates, mTOR kinase inhibitors only partially reduce proliferation of activated mature splenic B cells (Janes et al., 2010). Ongoing work in our laboratory is focused on resolving this paradox.

Another key question to be resolved is whether Akt in B cells has other important substrates besides FOXO transcription factors. The activation of PI3K and Akt has been linked to increased glycolysis in activated B cells (Doughty et al., 2006), but the relevant components linking Akt to metabolic changes have not been fully worked out. At the organismal level, Akt2 is primarily responsible for insulin/glucose homeostasis whereas Akt1 has a more dominant role in cell proliferation and differentiation (Fayard et al., 2010). Whether a similar division of labor occurs in B cells will be interesting to determine. It is also worth testing whether mTORC2 and its non-Akt substrates regulate B cell motility and chemotaxis, as reported in other systems (Masri et al., 2007; Gulhati et al., 2011). Identification of the Akt and mTOR substrates that control various aspects of B cell function could lead to novel targets for therapeutic intervention in immune diseases and cancer.

## Conflict of Interest Statement

The authors declare that the research was conducted in the absence of any commercial or financial relationships that could be construed as a potential conflict of interest.
